# Bilateral spontaneous simultaneous femoral neck occult fracture in a middle-aged man due to osteoporosis and vitamin D deficiency osteomalacia: A case report and literature review

**DOI:** 10.1016/j.ijscr.2019.06.058

**Published:** 2019-06-28

**Authors:** Kiyokazu Fukui, Ayumi Kaneuji, Hiroaki Hirata, Jun-ichi Tsujioka, Akihiro Shioya, Sohsuke Yamada, Norio Kawahara

**Affiliations:** a Department of Orthopedic Surgery, Kanazawa Medical University, Japan; b Department of Pathology and Medical Laboratory, Kanazawa Medical University, Japan

**Keywords:** Femoral neck fracture, Osteomalacia, Osteoporosis, Occult fracture

## Abstract

**Introduction:**

Bilateral stress fracture of the femoral neck is very rarely seen in healthy young patients who are neither athletes nor military recruits.

**Presentation of case:**

The present report describes a 51-year-old male patient, not an athlete and with no previous history of disease, who developed bilateral stress fracture of the femoral neck without displacement.

**Discussion:**

Simultaneous bilateral femoral neck fracture is a rare injury. In the present case, two factors predisposed to bilateral occult fracture of the femoral neck. The first was osteoporosis due to the patient’s smoking and alcohol abuse. The second was vitamin D deficiency osteomalacia associated with inadequate sun exposure.

**Conclusion:**

All patients who present with spontaneous hip pain should be evaluated for osteoporosis and osteomalacia and assessed for underlying occult fracture if they report groin pain or difficulty in walking, even when findings from plain X-ray are normal, to improve prognosis in this rare and serious condition.

## Highlights

• Physicians should take spontaneous femoral neck occult fracture into consideration if they report groin pain or difficulty in walking, even when findings from plain X-ray are normal.
• In a patient with spontaneous femoral neck occult fracture, diagnosing and treating the underlying etiology of osteoporosis and osteomalacia are essential for improving prognosis.
• This is the first report of a case of bilateral spontaneous simultaneous occult fracture of the femoral neck caused by osteoporosis and osteomalacia in a middle aged man.

## 1 Introduction

Several reports have addressed cases of adult femoral neck stress fracture, generally among athletes [1,2] and military recruits [3,4]. Such fractures tend to be uncommon in other (non-athlete non-military) adult populations, although there has been discussion of rare occurrences secondary to certain medical conditions including pregnancy, pelvic irradiation, corticosteroid exposure, chronic renal failure, and osteomalacia [5]. Here we report a rare case of bilateral spontaneous simultaneous stress fracture of the femoral neck in a 51-year-old male patient. The condition resulted from a combination of osteoporosis and vitamin D deficiency osteomalacia, which is a softening of the bones due to low levels of vitamin D. The patient was informed that data concerning the case would be submitted for publication, and he provided consent. This work has been reported in line with the SCARE criteria [6].

## 2 Case presentation

**Fig. 1:**
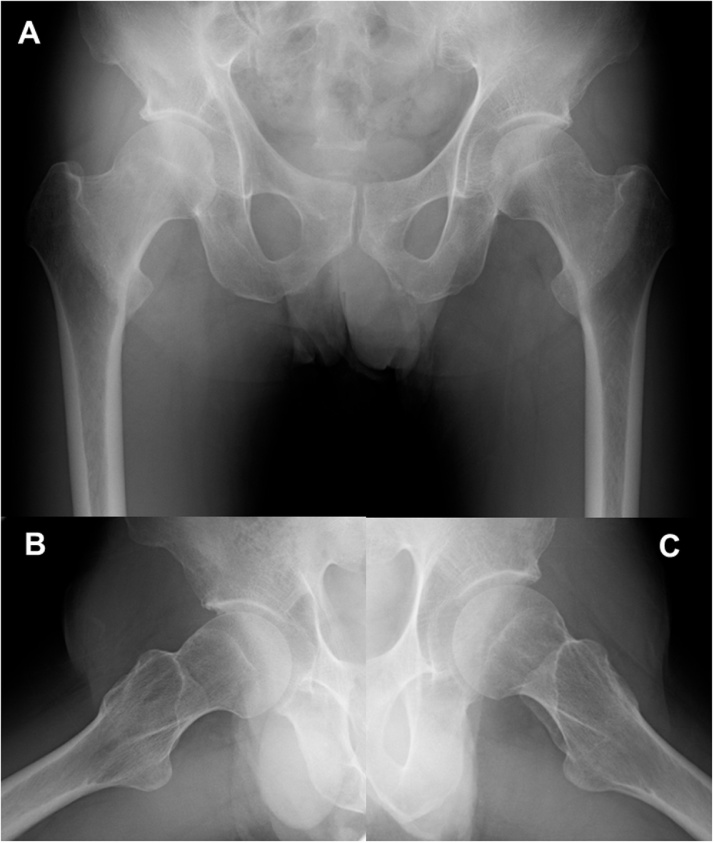
(A) Initial anteroposterior radiograph shows no significant findings except for mild pistol grip deformity on right hip and (B,C) small head-neck offset compared with left hip.

**Fig. 2:**
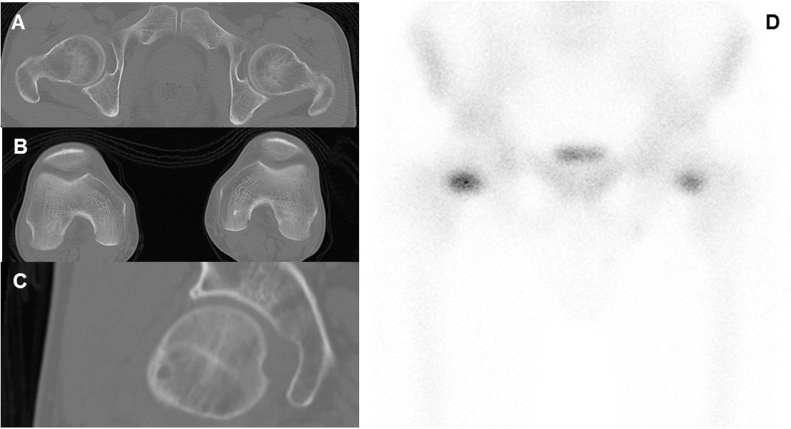
(A,B) Computed tomography axial planes indicate normal femoral neck anteversion on both hips, (C) multiplanar reconstruction shows herniation pit at lateral femoral head-neck junction on the right hip. (D) Bone scintigraphy scanning shows increased uptake in both femoral necks.

**Fig. 3:**
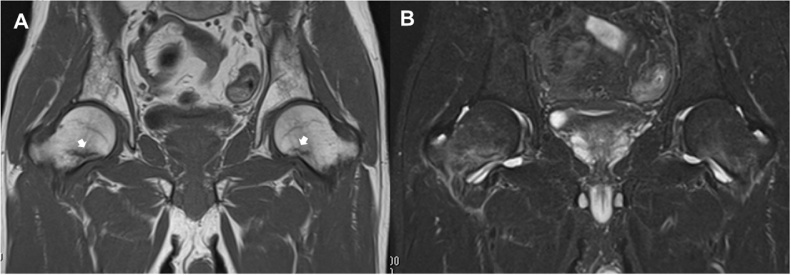
(A) T1 weighted MRI showing macroscopic fracture (white arrows). Fracture measures ≥50% of femoral neck width in coronal plane on right hip and <50% of femoral neck width in coronal plane on left hip. (B) MRI on short τ inversion recovery (STIR) sequence showing clear bone marrow edema in right femoral neck and slight bone marrow edema in left femoral neck.

A 51-year-old man who worked at a building maintenance service was referred to our university hospital due to spontaneous bilateral groin pain that had continued for 1 month. The first doctor suspected an injury to the hip adductor muscle. The patient appeared to be a healthy middle-aged man. He was not an athlete and had no history of metabolic disease, diabetes, rheumatoid arthritis, impaired renal function, or use of corticosteroids. He smoked and drank heavily, averaging 30 cigarettes and 2000 mL of beer daily. He worked nights, and thus had inadequate sun exposure for more than 5 consecutive years. He reported no falls or other trauma. Physical examination revealed pain with hip movements. The patient was 166 cm tall, weighed 55 kg, and has a body mass index of 20.0 kg/m^2^. At presentation, physical examination revealed no hip deformities. The range of motion was 120° in flexion, 10° in extension, 30° in abduction, 20° in adduction, 40° in external rotation, and 10° in internal rotation in each hip. Neurovascular findings were normal for both lower extremities. Standard radiographic findings were normal except for mild pistol grip deformity in the right hip (Fig. 1). In the right hip, femoral neck-shaft angle was 128° and femoral neck anteversion was 10°; those values were 127° and 9°, respectively, in the left hip. Computed tomography multiplanar reconstruction showed a herniation pit indicating cam-type femoroacetabular impingement at the lateral femoral head-neck junction (Fig. 2A–C). Bone scintigraphy showed increased uptake in both femoral necks, indicating possible stress fractures (Fig. 2D). Findings from magnetic resonance imaging (MRI) of the pelvis indicated fracture on the compression side of the distal portion of each femoral neck (Fig. 3). His laboratory studies showed increased alkaline phosphatase (ALP) activity (511 U/L; reference range: 115–359 U/L), normocalcemia (9.2 mg/dL), and hypophosphatemia (2.1 mg/dL). The level of 25-hydroxy-vitamin D (25-OH D) was 7.5 ng/mL (<20 ng/mL is defined as deficiency), and parathyroid hormone (PTH) was 560 pg/mL (reference range: 160–520 pg/mL), indicating osteomalacia. Low bone mineral density (BMD) was noted on dual-energy X-ray absorptiometry DXA (lumbar: 0.805 g/cm^2^; T score, −1.7; Z score, −1.2; right hip total: 0.498 g/cm^2^; T score, −2.9; Z score, −2.6; left hip total: 0.490 g/cm^2^; T score, −2.9; Z score, −2.6).

**Fig. 4:**
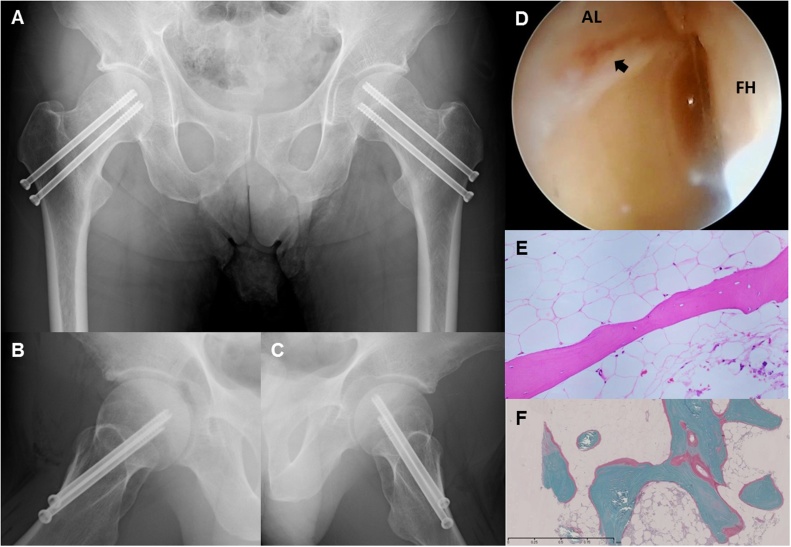
(A–C) Postoperative radiographs obtained immediate after bilateral internal fixation with cannulated screw. (D) Arthroscopic observation on the right hip showing cleavage at labro-cartilage junction and acetabular cartilage delamination (black arrow). AL: acetabular labrum, FH: femoral head (E) Photomicrograph obtained from bone marrow of right iliac crest. Thin bone trabeculae noted. (Hematoxylin and eosin stain) (F) Undecalcified bone section. Increase in unmineralized osteoid (pink stain) compared with mineralized bone (bluish-green stain). (Villanueva-Goldner stain).

We considered the patient’s past medical history, reviewed the risks and benefits of a surgical procedure [[7], [8], [9]], and decided to perform bilateral internal fixation with two 6.5-mm cannulated screws under general anesthesia (Fig. 4A–C). Because we wanted the patient to start walking with full weight-bearing as soon as possible after the surgery, we performed internal fixation on both the right hip (compression-sided fracture ≥50% of femoral neck width) and the left hip (compression-sided fracture <50% of femoral neck width). We received informed consent from the patient to perform hip arthroscopy on both hips to observe intra-articular pathology during the surgery and also to harvest bone marrow from iliac crest. Arthroscopic observation on the right hip showed typical pathological findings due to cam-type femoroacetabular impingement. The patient also had cleavage at the labro-cartilage junction and subsequent acetabular cartilage delamination classified as zone 2 grade 1 according to Konan’s classification [10] (Fig. 4D). Histopathological examination showed the bone trabeculae to be relatively thin, considering the patient’s male gender and age (Fig. 4E). The undecalcified bone section showed an increase of unmineralized osteoid, suggesting the presence of osteomalacia (Fig. 4F). There were no postoperative complications. The patient was prohibited from weight bearing for 1 week following surgery, after which full weight bearing was encouraged with two crutches. At discharge, 1 month after the surgery, the patient was free of pain and was able to bear his full weight without crutches. He received weekly alendronate sodium hydrate and adequate activated vitamin D (eldecalcitol 0.75 μg/day). One year after fracture treatment, he had no complaints. Laboratory tests showed normal levels of calcium, inorganic phosphorus, PTH, and 25-hidroxyvitamin D level.

## 3 Discussion

Simultaneous bilateral femoral neck fracture is a rare injury. To our knowledge, we are the first to describe a case of bilateral spontaneous simultaneous occult fracture of the femoral neck caused by osteoporosis and osteomalacia. This condition, although rare, can occur in the presence of osteomalacia, primary hyperparathyroidism renal osteodystrophy, and similar metabolic bone disease [11], and has also been associated with pregnancy, pelvic irradiation, and the use of corticosteroid and antacid drugs [5,11].

In the present case, two factors predisposed to bilateral occult fracture of the femoral neck. The first was osteoporosis due to the patient’s smoking and alcohol abuse, and the second was vitamin D deficiency osteomalacia associated with inadequate sun exposure. Tobacco and alcohol use have been associated with osteoporosis in men [12,13]; both bone formation and bone mass can be adversely affected by excessive drinking [14], and a study in male twins showed greater bone loss in smokers than in nonsmokers [15]. Vitamin D deficiency can be caused by a number of factors, including insufficient intake in food, inadequate exposure to sunlight, issues with the absorption of vitamin D, and conditions such as liver and kidney disorders that affect vitamin D catabolism.

This form of vitamin deficiency, which affects bone mineralization and results in softening of the bones, is termed “rickets” in children and “osteomalacia” in adults; femoral neck stress fracture from vitamin D deficiency has been reported in the literature [11,[16], [17], [18], [19], [20]]. Insufficient exposure to sunlight is the most important factor in low serum vitamin D and a common cause of osteomalacia, since more than 90% of vitamin D is obtained from ultraviolet B light and the remainder comes from the diet [21]. This particularly affects the elderly, who tend to be confined indoors with low levels of activity and poor diet, usually resulting in at least a subclinical deficiency of vitamin D [22]. Such subclinical deficiency will commonly progress silently, without obvious clinical manifestations, until it is suddenly revealed when a spontaneous fracture occurs. The literature suggests that patients usually complain of bilateral groin or thigh pain that persists over a period of a few weeks to several months [[23], [24], [25], [26], [27], [28], [29]], so that these fractures develop gradually and diagnosis is typically delayed. In cases such as this, even if the initial radiographs are negative, the possibility of bilateral hip fracture should be considered, particularly if the pain worsens suddenly or the patient exhibits signs of bone insufficiency [26]. Although a previous study on radiographic features of femoroacetabular impingement in femoral neck stress fracture showed a relatively high incidence of pincer type morphology [30], our arthroscopic observation indicated cleavage at the labro-cartilage junction due to cam-type impingement (Fig. 4D). Uchida et al. demonstrated that labral tears associated with underlying bone abnormality can lead to micro-instability, a result of concurrent pathologies including cartilage damage and subchondral insufficiency fracture (SIF) of the femoral head, and suggested that it is conceivable that labral tears may contribute to the pathomechanism of SIF of the femoral head [31]. However, if that pathomechanism were associated with a labral injury, the insufficiency fracture should have occurred at the subchondral region of the femoral head, rather than at the femoral neck as in the current case. Earlier research in our department suggested the possible involvement of the inverted acetabular labrum in subchondral insufficiency fracture associated with subsequent rapidly destructive hip OA [[32], [33], [34], [35]]. Our current findings show that acetabular labral injury alone is not enough to cause SIF of the femoral head in patients with fragile bone quality, and indicates that another factor such as inversion of the acetabular labrum might be required to produce SIF of the femoral head rather than fracture of the femoral neck. Regardless of age, patients who present with spontaneous hip pain should be checked for serum vitamin D, ALP levels, and BMD, should be evaluated for osteoporosis and osteomalacia, and should be queried in detail about their patient background. A bilateral hip MRI or bone scan is needed to assess for underlying occult fracture if patients report groin pain or difficulty in walking, even when findings from plain X-ray are normal. Diagnosing and treating the underlying etiology of osteoporosis and osteomalacia are essential for improving prognosis in this rare and serious condition.

## 4 Conclusion

Physicians should take spontaneous femoral neck occult fracture into consideration if they report groin pain or difficulty in walking, even when findings from plain X-ray are normal. In a patient with spontaneous femoral neck occult fracture, diagnosing and treating the underlying etiology of osteoporosis and osteomalacia are essential for improving prognosis.

## Conflicts of interest

None.

## Funding

None.

## Ethical approval

Case reports are exempt from the need of IRP approval in our institute.

## Consent

Written informed consent was obtained from the patient for publication of this case report and accompanying images. A copy of the written consent is available for review by the Editor-in-Chief of this journal on request.

## Author’s contribution

Conceptualization, Writing of manuscript, Literature review: Kiyokazu Fukui.

Data collections: Kiyokazu Fukui, Hiroaki Hirata, Jun-ichi Tsujioka, Akihiro Shioya.

Data analysis: Kiyokazu Fukui, Sohsuke Yamada.

Reviewing of the final version of the manuscript: Kiyokazu Fukui, Ayumi Kaneuji, Norio Kawahara.

## Registration of research studies

Not applicable.

## Guarantor

Kiyokazu Fukui.

## Provenance and peer review

Not commissioned, externally peer-reviewed.
